# Amplified Detection of the Aptamer–Vanillin Complex with the Use of Bsm DNA Polymerase

**DOI:** 10.3390/s18010049

**Published:** 2017-12-26

**Authors:** Mariia Andrianova, Natalia Komarova, Vitaliy Grudtsov, Evgeniy Kuznetsov, Alexander Kuznetsov

**Affiliations:** Scientific-Manufacturing Complex Technological Centre, 1–7 Shokin Square, Zelenograd, 124498 Moscow, Russia; nat.v.kom@gmail.com (N.K.); vitaliycatgrudcov@gmail.com (V.G.); kev@tcen.ru (E.K.); kae@tcen.ru (A.K.)

**Keywords:** aptasensor, aptamer, signal amplification, Bsm DNA polymerase, ISFET, vanillin

## Abstract

The electrochemical detection of interactions between aptamers and low-molecular-weight targets often lacks sensitivity. Signal amplification improves the detection of the aptamer-analyte complex; Bsm DNA polymerase was used to amplify the signal from the interaction of vanillin and its aptamer named Van_74 on an ion-sensitive field-effect transistor (ISFET)-based biosensor. The aptamer was immobilized on the ISFET sensitive surface. A short DNA probe was hybridized with the aptamer and dissociated from it upon vanillin addition. A free probe interacted with a special DNA molecular beacon initiated the Bsm DNA polymerase reaction that was detected by ISFET. A buffer solution suitable for both aptamer action and Bsm DNA polymerase activity was determined. The ISFET was shown to detect the Bsm DNA polymerase reaction under the selected conditions. Vanillin at different concentrations (1 × 10^−6^–1 × 10^−8^ M) was detected using the biosensor with signal amplification. The developed detection system allowed for the determination of vanillin, starting at a 10^−8^ M concentration. Application of the Bsm DNA polymerase resulted in a 15.5 times lower LoD when compared to the biosensor without signal amplification (10.1007/s00604-017-2586-4).

## 1. Introduction

Aptamers are typically, single-stranded oligonucleotide (DNA or RNA) molecules <100-bp that can bind to other molecules with high specificity and affinity. Aptamers have been generated against a wide variety of targets including small molecules such as metal ions [1], aminoglycoside antibiotics [2], cocaine [3], adenosine [4] and theophylline [5]. The selective binding abilities of aptamers were employed to develop different sensors [6,7]. A variety of aptamer-based sensors for low-molecular-weight substrates have been developed recently including electrochemical [8,9,10,11] and optical [12,13,14]. 

Electrochemical aptasensors are one of the main types of sensors based on aptamers, especially for low-weight targets. The main feature of electrochemical sensors is the use of different labels and enhancements to amplify the signal since the signal from the interaction of the target with the aptamer is rather weak. Different modifications of aptamers and labels are used to amplify biosensing events. The main amplification approaches use nanoparticles [15], quantum dots [16,17], DNAzymes [18], a ferrocene redox probe [9], modification with methylene blue [19,20], and fluorescent ligands [21].

The field effect transistor (FET)-based sensor is a type of electrochemical sensor, which can be considered as an integrated device containing a receptor layer (aptamer) that selectively recognizes and binds the target compound and a signal transducer, and represented by an ion-selective field effect transistor (ISFET) that registers the recognition [22]. ISFET sensors exhibit significant advantages over other signal transducers including low manufacturing costs have been described in the literature. The label-free and reagentless aptamer-based sensor for adenosine allows for the detection of 5 × 10^−5^ M of adenosine [23]. The detection scheme was based on the dehybridization of a DNA probe upon the addition of adenosine to an aptamer-complement probe system. Furthermore, a sensor for cocaine with Au nanoparticles as the amplifying labels was developed [24]. The limit for the detection of cocaine was 1 × 10^−6^ M. Additionally, there is an aptamer-modified silicon nanowire field-effect transistor for K^+^ [25], and a biosensor for adenosine triphosphate based on a hairpin DNA aptamer coupled with a field-effect transistor has been described in [26]. 

In this work, a new chemical amplification approach for the detection of aptamer-vanillin interaction on the ISFET with the use of Bsm DNA polymerase reaction is presented. The key moment of amplification is in the enzymatic reaction, which is initiated after the hybridized probe leaves the transistor surface with the aptamer upon the reaction of the aptamer and the target. The principle of Bsm DNA polymerase reaction was taken from [27] and adapted for measurements with the selected aptamer for vanillin. The aptamer for the low-molecular-weight target (vanillin) was previously obtained by us using a Capture-SELEX technique [28]. To conclude, an electrochemical biosensor with amplification consisting of the signal transducer (ISFET), recognition layer (aptamer for vanillin), and reaction solution (Bsm DNA polymerase reaction mixture) was developed during this work. This allowed us to decrease the limit of detection of vanillin by 15.5 times in comparison with the biosensor without amplification [28].

## 2. Materials and Methods 

### 2.1. Materials 

Bsm DNA polymerase, large fragment, Bsm buffer, dNTP solution, TBE electrophoresis buffer, and SYBR Gold dye were all purchased from Thermo Fisher Scientific (Waltham, MA, USA). Vanillin and 3-aminopropyltriethoxysilane were from Sigma Aldrich (St. Louis, MO, USA). Azidobutyric NHS ester and Cu(II)-TBTA reagent were obtained from Lumiprobe (Moscow, Russia). Streptavidin-coated magnetic beads were supplied by New England Biolabs (Ipswich, MA, USA). All ssDNA sequences (Supplementary Material, Table S1) were synthesized by DNA Synthesis Ltd. (Moscow, Russia). The following buffers were used:

Selection buffer: 20 mM Tris-HCl (pH 7.6), 100 mM NaCl, 5 mM KCl, 2 mM MgCl_2_, 1 mM CaCl_2_.

Low molarity selection buffer: 2 mM Tris-HCl (pH 7.3), 5 mM NaCl, 1 mM KCl, 1 mM MgCl_2_, 0.5 mM CaCl_2_.

Bsm buffer: 20 mM Tris-HCl (pH 8.8), 10 mM KCl, 10 mM (NH_4_)_2_SO_4_, 2 mM MgSO_4_, 0.1% (*v*/*v*) Tween 20.

Wash/Binding Buffer: 0.5 M NaCl, 20 mM Tris-HCl (pH 7.5), 1 mM ethylenediaminetetraacetic acid.

### 2.2. ISFET Fabrication 

An n-type ISFET was fabricated using standard 1.2 µm FD SOI CMOS technology at the SMC Technological Centre, Zelenograd, Russia [29]. The gate dielectric consisted of 10 nm SiO_2_ covered by 100 nm Ta_2_O_5_, (thermal oxidation of PVD tantalum film at 850 °C in O_2_, 10 min). The transistor channel size was 100 × 6 µm.

### 2.3. Fluorescence Measurement of Bsm DNA Polymerase Reaction

The amount of Cy3-labelled DNA was monitored by Cy3 fluorescence. Measurements were carried out on a Tecan Infinite M200 microplate spectrofluorimeter (Tecan, Zurich, Switzerland) using excitation at 540 nm and emission at 570 nm.

### 2.4. Aptamer Selection

The aptamer for vanillin was selected from the ssDNA library according to the Capture-SELEX procedure [2] in the SMC Technological Centre. Two mM of vanillin was used as the target for selection. B1_biotin was used as the capture probe and B1_bank as the initial ssDNA library. The B1_bank consisted of two primer binding sites of 18 and 19 nt, and a random fragment of 50 nt, which was separated by capture-sequencing of 12 nt (complementary to B1_biotin) to 10 and 40 nt. The selected aptamer for vanillin has the sequence 5′-CGACCAGCTCATTCCTCAGGAGAAACATGGA GTCTCGATGATAGTAGGAGCGGCGGAACGTAGGAAGAGAGGATGACGGAGGATCCGAGCTCACCAGTC-3′. The name of the aptamer is Van_74, according to the sequencing of selected aptamers (among 83 sequences) [28].

### 2.5. Aptamer Immobilization

Immobilization of the aptamer on the gate surface was conducted via “click-chemistry” in three stages [22]: (1) 3% aminopropyltrietoxysilane in methanol for 30 min, (2) 4.4–6.0 mM azidobutyric NHS ester in 0.1 M Tris/HCl, pH 8.65 for 4 h, (3) 5 × 10^−7^ M alkyne-modified aptamer with 0.05 mM copper-containing reagent (Cu(II)-TBTA) and 2 mM ascorbic acid in the same buffer overnight. After aptamer immobilization, the aptamer was hybridized with short complementary probes for 2 h.

### 2.6. ISFET Measurements

The operating subthreshold mode of the ISFET was determined from the I-V_G_ curves (V_DS_ = 0.1 V); the threshold voltage V_t_ and subthreshold slope S were used. Time-dependent changes in the current I_DS_ (V_G_ = const) were measured and the surface potential change $∆\varphi_{i}\approx \;\frac{S}{S_{0}}n\varphi t\frac{I_{i+1}-I_{i}}{I_{i}}$ was used as a signal. All measurements were carried out in a well-like structure made of organic ink deposited onto the surface of the crystal with a pneumatic dispenser Nordson EFD Ultimus V pneumatic dispenser (Westlake, OH, USA; 5.7 bar at 22 °C) and Janome JR2303 robot positioning system (Tokyo, Japan) [30]. Time-dependent changes of I_DS_ (V_G_ = const) were recorded in a well-like structure containing 25–30 μL of buffer and during the record 1–1.5 μL of target was added.

### 2.7. Aptamer Characterization by PAGE *[28]*

Washed streptavidin-coated magnetic beads were incubated with the capture probe B1_biotin (100 μL of beads (c = 4 mg/mL): 50 μL of Wash/Binding Buffer with 400 pmol of B1_biotin) for 2 h. After incubation, the beads were well washed in the Selection buffer and incubated with the aptamer (50 μL of the Selection buffer with 800 pmol of aptamer) overnight with interval mixing (1200 rpm for 30 s, 9 min 30 s without mixing) using an Eppendorf Thermomixer Comfort (Hamburg, Germany). The unbound ssDNA was washed away by rinsing the particles with the SB seven times. Weakly bound sequences were removed by incubating the particles at 28 °C for 15 min, followed by seven washes with SB.

Modified magnetic beads were added to the buffer containing vanillin, and incubated for 1 h at 21 °C with constant mixing at 850 rpm (5 μL of beads for each sample of the total volume of 50 μL of beads). Aliquots of 5 μL from the washout were used in PCR (20 μL of PCR premix for each sample) and the eluted aptamer was amplified. The reaction mixture contained 1.0 µM of primers, 0.2 mM MgCl_2_, 0.25 mM of each dNTP, and 0.06 U/µL Taq DNA polymerase in 75 mM Tris-HCl (pH 8.8 at 25 °C), 20 mM (NH_4_)_2_SO_4_, and 0.01% (*v*/*v*) Tween 20. Amplification was carried out in a Tercyk Thermocycler (Moscow, Russia) and included an initial denaturation step at 95 °C for 5 min; 12 cycles of 95 °C for 30 s, 66 °C for 30 s, and 72 °C for 30 s; and a final extension step at 72 °C for 2 min.

Five μL of the PCR product without purification were loaded to the nondenaturing PAGE. After 1 h of separation at 120 V in a Mini Protein Tetra Cell System (Bio-Rad, Hercules, CA, USA), the gel was stained with SYBR Gold and scanned by a Bio-1000F scanner (Microtek, Hsinchu, Taiwan). The GelQuant.NET software (Biochemlabsolutions.com, University of California, San Francisco, CA, USA) was used to analyze the intensity of DNA strips in the gel.

## 3. Results

**Figure 1:**
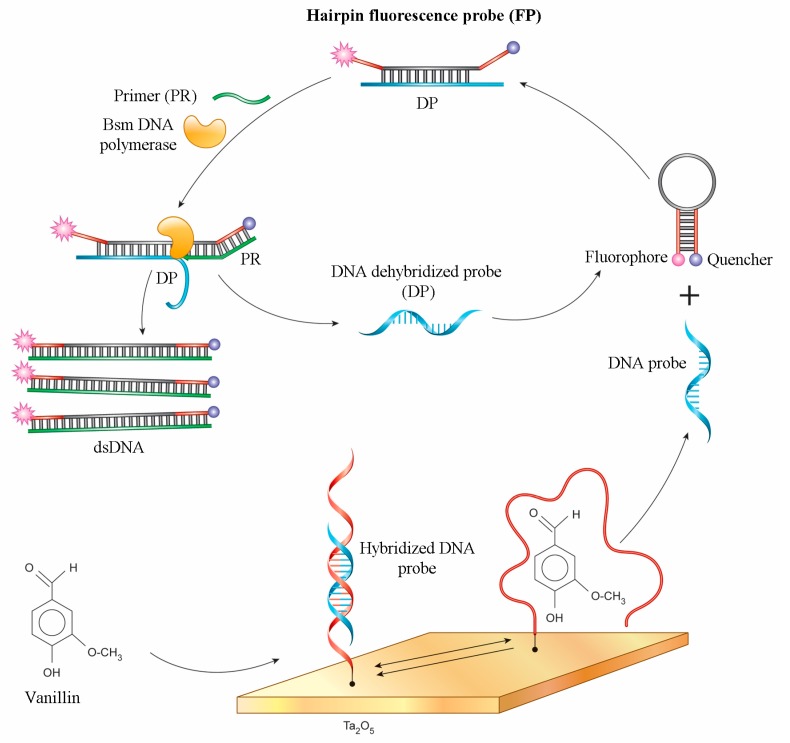
Detection scheme: release of the dehybridized DNA probe during aptamer-vanillin complex formation and the Bsm DNA polymerase reaction with probe detected by ISFET.

The scheme assumed that the immobilized aptamer on the ISFET surface was hybridized with the DNA probe, which was released from the aptamer during the addition of vanillin (Figure 1). This dehybridized DNA probe then participated in the Bsm DNA polymerase reaction which was detected by the ISFET. The main components of this reaction were a hairpin fluorescence probe (FP), a short primer (PR), and a dehybridized DNA probe (DP), which can hybridize with FP and open the hairpin. After that, the hairpin annealed with the primer and triggered the polymerization reaction [27]. Polymerase has a strong strand displacement activity and lacks exonuclease activities, so the DP was displaced and hybridized to another FP. Therefore, this was the amplification of the signal at a low concentration of DP. Amplification was monitored in situ with the use of a highly sensitive ISFET (Figure 1).

### 3.1. Optimization of Operating Conditions

The idea of amplifying the signal from a small amount of ssDNA with the use of Bsm DNA polymerase was taken from [27]. However, FP, DP and PR were designed corresponding to the aptamer sequence (Table 1).

The aptamer, Van_74, was developed using the capture-SELEX protocol [2], which implied a capture-sequence in its structure that was used for ssDNA library fixation onto the solid support via hybridization with the capture-probe B1_biotin. So, part of the designed DP contained sequence of the capture-probe B1_biotin, which was complementary to the capture-sequence of Van_74, to ensure its dehybridization upon the addition of vanillin. The other nucleotides in the DP sequence were optimized to non-complement the Van_74 sequence and partly complement the FP to break down its hairpin. Moreover, the DP and FP were designed to ensure the single possible stem-loop structure for the FP predicted by the Mfold web server [31] with free energy of −13.35 kJ at 21 °C [32] (Figure S1). The nucleotide sequence for the stem was analogous to the probe used in [27], and the loop part was complementary to the DP sequence. The primer sequence was the same as in the original paper [27].

Initially, the conditions under which detection will take place were optimized before the fabrication of the sensor. It is known that an aptamer can form an effective complex with a target only under specific conditions (buffer composition, salt concentration, pH, temperature), which mostly correspond to the conditions of the aptamer selection process [33,34,35,36]. The aptamer for vanillin presented in this work was selected in the Selection buffer with a total molarity of 128 mM (pH 7.6) and at the temperature of 21 °C. However, according to supplier information, Bsm DNA polymerase is active in a wide range of temperatures from 30 to 63 °C (with an optimum activity at 60 °C) and should be used in Bsm buffer with a total molarity of 42 mM (pH 8.8) and content of surfactant (Tween 20).

**Figure 2:**
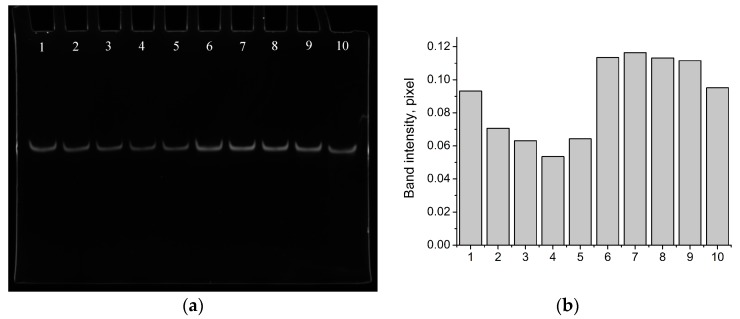
Nondenaturating PAGE (photo without any modification) of the PCR amplified (16 cycles) washout probes from the magnetic beads modified with B1/Van_74 (**a**) and image analysis of phoresis with GelQuant.NET (**b**) for different incubation buffers: (1) Selection buffer with vanillin (1.5 mM); (2) Selection buffer with vanillin (1.5 mM), repeat; (3) Selection buffer; (4) Selection buffer, repeat; (5) Selection buffer, repeat; (6) Bsm buffer with vanillin (1.5 mM); (7) Bsm buffer with vanillin (1.5 mM), repeat; (8) Bsm buffer; (9) Bsm buffer, repeat; and (10) Bsm buffer, repeat.

It was shown that the aptamer did not bind vanillin in the Bsm buffer (Figure 2 and Figure S2). For this purpose, magnetic beads coated with the aptamer (via B1) were incubated in vanillin solutions (1.5 mM) in the Bsm buffer and the Selection buffer. PCR amplification of the washout showed that the signal (band intensity) from the Bsm buffer with vanillin had no significant difference from the background (Figure 2 and Figure S2). On the other hand, incubation of the modified magnetic beads in the Selection buffer with vanillin showed the formation of the aptamer-vanillin complex (Figure 2 and Figure S2).

**Figure 3:**
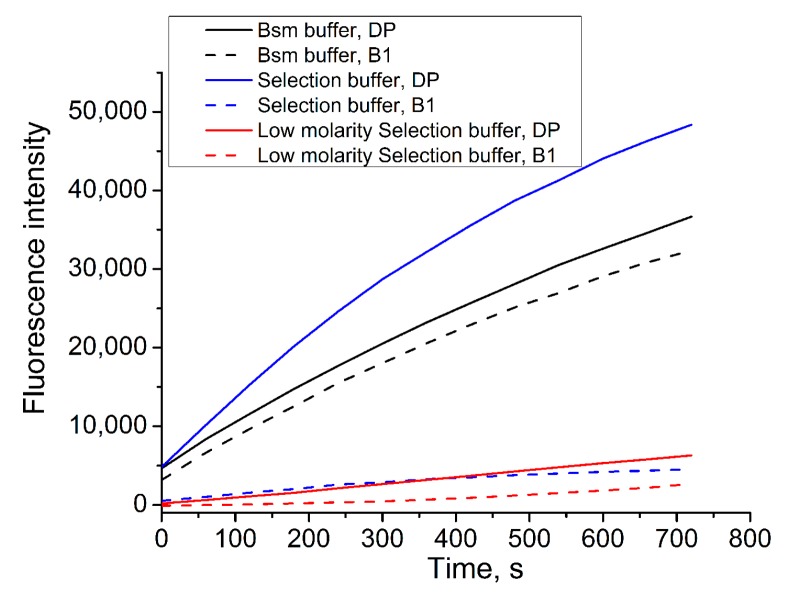
Kinetics of Bsm DNA polymerase reaction (after subtraction of the background) monitored by spectrofluorimeter in different buffers and probes. Conditions: buffer, 0.2 mM dNTP, 0.05 pmol/μL FP, 1.6 pmol/μL PR, 0.1 U/μL BSM DNA polymerase, 0.1 pmol/μL DP/B1.

Thus, the choice of working buffer was essential for several reasons. It had an impact on aptamer behavior, the Bsm DNA polymerase reaction, and the ISFET sensitivity. The sensitivity of ISFET greatly depends on the distance of charges from the interface, the so-called Debye length, beyond which potential change cannot be properly detected [37]. The Debye length is a function of electrolyte concentration [38]. Thus, the choice of a buffer with low molarity was preferable. Previously, it has been shown that aptamers can bind vanillin not only in the Selection buffer, but in the low molarity selection buffer [28]. Thus, the investigation of the Bsm DNA polymerase reaction in the low molarity selection buffer was undertaken (Figure 3).

As seen in Figure 3, the maximum Bsm DNA polymerase activity corresponded to the Selection buffer and DP and the minimum to low molarity Selection buffer and B1. In general, the polymerase activity was greatest when using DP as a probe compared to B1. This behavior was obviously due to the length of the probe and the interaction with FP. Since the DP was longer than B1 by 14 bp and was complementary to FP at least by 6 bp more, it was more likely to open the hairpin of the FT, which prevents the polymerase reaction. Therefore, we decided to use a DP probe in the low molarity buffer on the ISFET for the Bsm DNA polymerase reaction. 

**Figure 4:**
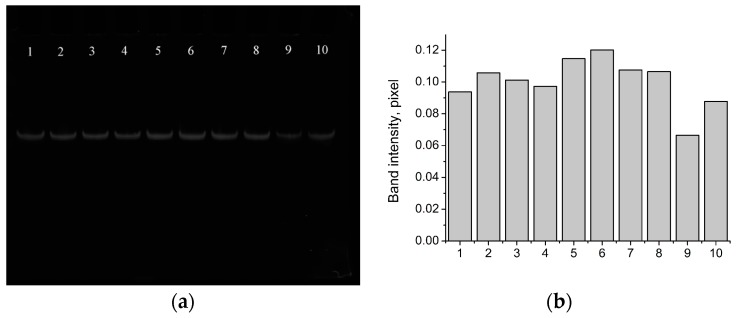
Nondenaturating PAGE (photo without any modification) of PCR amplified (10 cycles) washout probes from magnetic beads modified with DP/Van_74 (**a**) and image analysis of phoresis with GelQuant.NET (**b**) for different vanillin concentrations in low molarity Selection buffer: (1) 1 × 10^−3^ M; (2) 1 × 10^−3^ M, repeat; (3) 2.5 × 10^−4^ M; (4) 2.5 × 10^−4^ M, repeat; (5) 6.25 × 10^−5^ M; (6) 6.25 × 10^−5^ M, repeat; (7) 1.6 × 10^−5^ M; (8) 1.6 × 10^−5^ M, repeat; (9) low molarity selection buffer; and (10) low molarity selection buffer, repeat.

For this, the DP probe was tested as a dehybridization probe from aptamer Van_74 during the addition of vanillin. For this purpose, nondenaturating PAGE of washouts from the modified magnetic beads was carried out (Figure 4 and Figure S3). The obtained results showed that the B1 probe could be changed by DP in the experiments with the aptamer and target: the complementary between DP and Van_74 could be broken during the vanillin addition. 

To sum up, the obtained results presented in this section are:

- Aptamer for vanillin could not perform in the Bsm buffer and could bind vanillin in the Selection buffer and low molarity selection buffer.
- Bsm DNA polymerase is active in a low molarity selection buffer and the greatest activity is with DP compared to B1.
- B1 probe can be replaced by DP as a dehybridization probe from the aptamer Van_74 during the vanillin addition.

### 3.2. Bsm DNA Polymerase Reaction on the ISFET

**Figure 5:**
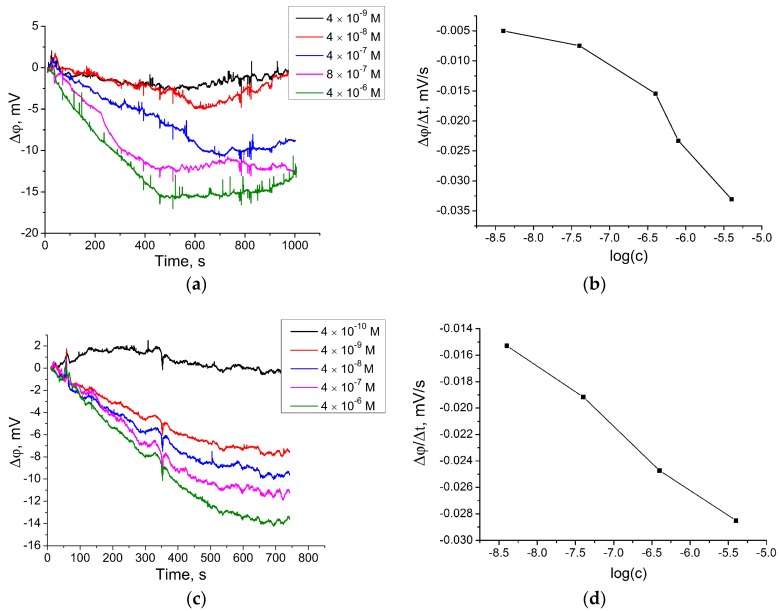
(**a**) Real time signal of the ISFET (in ∆ϕ, with background subtraction) during the Bsm DNA polymerase reaction in homogenous solution in low molarity selection buffer initiated (30–40 s) by the addition of DP at different concentrations (final concentration is marked on the picture); (**b**) Slope (∆ϕ/∆t) dependence on DP concentration calculated from real time signal curves. Reaction conditions: 0.2 mM dNTP, 0.05 pmol/μL FP, 1.6 pmol/μL PR, 0.1 U/μL BSM DNA polymerase; (**c**) Real time signal of the ISFET (in ∆ϕ, with background subtraction) during the Bsm DNA polymerase reaction in homogenous solution in low molarity selection buffer initiated (30–40 s) by the addition of DP at different concentrations (final concentration is marked on the picture); (**d**) Slope (∆ϕ/∆t) dependence on DP concentration calculated from real time signal curves. Reaction conditions: 0.2 mM dNTP, 1.0 pmol/μL FP, 1.6 pmol/μL PR, 0.1 U/μL BSM DNA polymerase.

The response of the ISFET to the Bsm DNA polymerase reaction in the homogenous solution was tested. A well-like structure made of organic ink on the ISFET surface was filled with reaction solution (low molarity selection buffer, 0.2 mM dNTP, 0.05 pmol/μL FP, 1.6 pmol/μL PR, 0.1 U/μL BSM DNA polymerase) except the DP. Then, different concentrations of DP were added to the well while the drain current (I_DS_) induced by the reaction was measured, and then, according to the I_DS_ surface potential change, was calculated. The decrease in surface potential after the addition of the DP was proportional to its concentration (Figure 5a). The main criteria for the reaction proceeding was the slope (mV/s) after DP addition (Figure 5b). Thus, the limit of detection (LoD) of DP on the ISFET in low molarity selection buffer was 4.0 × 10^−9^ M. 

The same reaction, but with a higher concentration of FP (1 pmol/μL) is given on Figure 5c,d. The quantity of FP is important for the amplification reaction as the Bsm DNA polymerase can act until all single stranded FP become double stranded DNA. Thus, the quantity of FP should be greater than DP so the amplification of the signal from DP can be seen (FP >> DP). Therefore, it was shown that am increase in FP concentration led to the enhancement of the signal from low concentrations of DP. The LoD of DP on the ISFET in these conditions was 4.0 × 10^−9^ M. The Bsm DNA polymerase reaction on the ISFET in Bsm buffer and Selection buffer is presented in the Supplementary Material (Figures S4 and S5). It was shown that in the BSM buffer, the reaction proceeded faster than in the low molarity selection buffer and could be detected by the ISFET. The polymerase reaction in the Selection buffer was not detected by the ISFET at the applied concentration of DP. Furthermore, the Bsm DNA polymerase reaction on the ISFET modified with Van_74/probe DP in the low molarity selection buffer was held. This experiment was supposed to show the response of the modified ISFET, where the sensitive surface carried negative charge due to DNA, to the Bsm DNA polymerase. It turned out that the direction of the surface potential change remained the same as for nonmodified ISFET surface (Figure S6).

### 3.3. Amplified Detection of the Aptamer–Vanillin Complex with the Bsm DNA Polymerase and ISFET

**Figure 6:**
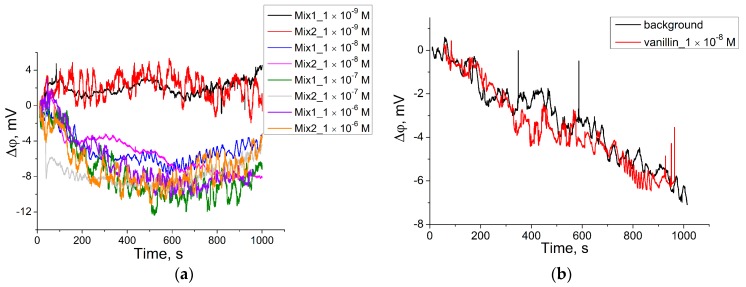
(**a**) Dependence of response (with background subtraction) of modified with Van_74/DP ISFETs to the Bsm DNA polymerase reaction with the addition of different vanillin concentrations. Conditions: **mix1**—low molarity selection buffer, 0.2 M;M dNTP, FP 0.05 pmol/μL, PR 1.6 pmol/μL, Bsm DNA polymerase 0.1 U/μL; **mix2**—low molarity selection buffer, 0.2 MM dNTP, FP 1.0 pmol/μL, PR 1.6 pmol/μL, Bsm DNA polymerase 0.1 U/μL, T = 22 °C; (**b**) Real time signal of the ISFET (in ∆ϕ) of modified with Van_74/DP ISFETs to the addition of vanillin (final concentration 1 × 10^−8^) concentration. Conditions: low molarity selection buffer, 0.2 MM dNTP, PR 1.6 pmol/μL, Bsm DNA polymerase 0.1 U/μL.

The aptamer for vanillin was immobilized on the ISFETs surface and hybridized with DP. Aliquots with different concentrations of vanillin were added to a well-like structure onto the ISFETs surface containing the Bsm DNA polymerase reaction mixture. For the measurements, two reaction mixtures were used; the difference between them was in the concentration of FP. The concentration of FP in Mixture 1 was 0.05 pmol/μL, and Mixture 2 was 1 pmol/μL, 20 times higher (Figure 6). The addition of vanillin to the system in concentrations from 1 × 10^−6^ to 1 × 10^−8^ M caused an equally significant decrease in the surface potential change. The signal from the addition of vanillin in a concentration of 1 × 10^−9^ M did not significantly differ from the background. Additionally, the signal from only the dehybridization of DP during the vanillin addition in the reaction condition did not differ from the background (Figure 6b), which testifies in favor of the amplification technique.

The proposed approach allowed us to conduct a qualitative analysis of vanillin (not less than 1 × 10^−8^ M). Compared with our previous research [29], the limit of detection of vanillin during this research was increased by 15.5 times (Table 2). The possibility to carry out only qualitative analysis and not quantitative can be explained by the time required for reaction proceeding: diffusion of vanillin to the ISFET surface, the interaction between the aptamer and vanillin, and the dehybridization of DP, initiation of the Bsm reaction, etc. The reaction time in the experiments was limited due to evaporation in the open well-like system. The use of a microfluidic system may solve this problem in future.

The fabricated biosensor had good reproducibility and stability. The relative standard deviation (RSD) of the biosensor response to 10^−8^ M of vanillin was approximately 7% for five successive measurements. In the practical performance of the sensor, approximately 500 s of exposure time was required for each measurement.

The main mechanism of ISFET sensitivity is determined by the surface potential change at the interface of surface/solution. This surface potential change comes from a change in the electrochemical potential of electrons in the solution during the enzymatic reaction and change in the quantity of DNA probes adsorbed on the ISFET surface. The electrochemical potential of the solution is the summary of the electrochemical potentials of all components in the system: buffer reagents, salts, enzyme, and substrates. During the enzymatic reaction, the total electrochemical potential of the solution changes due to local pH change and this change causes a change in the surface potential, which is detected by the ISFET. At low concentrations of vanillin (and, by extension, low concentration of desorbed DP) the ISFET is more sensitive to the reaction when compared to probe dehybridization (desorption from the surface), which is the difference in the values of electrochemical potential connected to each process. Local pH change is due to the release of proton (H^+^) during the polymerase reaction: the phosphodiester bond is formed between the α-phosphate of the incoming dNTP and 3′-OH group of deoxyribose, the chemical reaction generates a pyrophosphate and proton molecule [39].

## 4. Discussion

The rapid development of microelectronic technologies in the last few decades has led to the fact that CMOS technology has now become a reliable and inexpensive technological platform for creating electrochemical sensors. This has led to the appearance of miniature biosensors on silicon chips. In contrast to conventional biosensors, CMOS biosensors have a low cost, are portable, can easily integrate into more complex systems, and have low energy consumption. In combination with aptamers, CMOS biosensors can serve as a promising universal platform for creating diagnostic systems. However, one of the drawbacks for wide use of such a platform is the low detection limits of low-weight molecules (see Table 2). One of the potential solutions to increase the detection limit is to use chemical amplification of the signal.

In comparison with other FET sensors presented in Table 2, the obtained sensor showed equal or better performance in the determination of the small molecule, particularly in, vanillin. It should be noted that the limit of detection of the protein molecules was lower in comparison to low-weight molecules because more charge was introduced into the system that resulted in a larger disturbance of electromagnetic field near the sensitive surface.

Despite the promising results demonstrated in this study, the use of the Bsm DNA polymerase reaction as the amplification technique has some limitations. In this research, only a qualitative analysis of vanillin was shown. This drawback can be overcome by the use of a microfluidic system. One more limitation is to select a working buffer where both the aptamer and the enzyme can work. Despite these limitations, the use of the Bsm DNA polymerase reaction with the combination of ISFET and aptamer showed successful results in the amplification of the signal from the interaction of aptamer Van_74 and small molecule vanillin.

## 5. Conclusions

The ISFET-based biosensor to detect the interaction between the aptamer and small target—vanillin—using the Bsm DNA polymerase reaction as a chemical amplification was developed. It allowed us to carry out qualitative analysis of vanillin and improve the LoD of vanillin by 15.5 times when compared to a biosensor without amplification. 

Further improvements of the biosensor are planned by fabricating a microfluidic system on the surface of the chip. This avoids the evaporation of reagents and minimizes the signal-to-noise ratio.

## Figures and Tables

**Table 1:** Oligonucleotide sequences used in Bsm DNA polymerase reaction. The underlined sequence is a capture sequence.

Sequence Name	Sequence, 5′-3′ Direction	Comment
Van_74	CGACCAGCTCATTCCTCAGGAGAAACATGGAGTCTCGATGATAGTAGGAGCGGCGGA ACGTAGGAAGAGAGGATGACGGAGGATCCGAGCTCACCAGTC	Aptamer for vanillin
B1	CATCGAGACTCC	Capture probe without biotin
DP	ACCACATCGAGACTCCTGTGTCCTTT	Bsm dehybridization probe
FP	FAM-TCTTGGFCFCAGGAGTCTCGATGTGGTATTGTGTCCAAGA-BHQ1	Fluorescence probe
PR	TCTTGGAC	Primer

**Table 2:** Sensors based on FETs and aptamers presented in the literature.

Substance	LoD, M	Type	Reference
Low-weight molecules			
Adenosine	5 × 10^−5^	Si-ISFET	[23]
Adenosine	1 × 10^−11^	Graphene-FET	[40]
Cocaine	1 × 10^−6^	Si-ISFET	[24]
K^+^	K_ass_ = (2.18 ± 0.44) × 10^6^	Si-ISFET	[25]
Bisphenol A	1 × 10^−12^–1 × 10^−14^	Carbon-FET	[41]
ATP	-	FET	[26]
Vanillin (our previous work)	1.55 × 10^−7^	Si-ISFET	[28]
Vanillin	1 × 10^−8^	Si-ISFET	This work
Protein molecules			
Thrombin	2.5 × 10^−8^	Si-ISFET	[42]
Thrombin	7 × 10^−7^	Si-ISFET	[43]
Thrombin	5 × 10^−8^	Polypyrrole-FET	[44]
Thrombin	2 × 10^−11^	Carbon-FET	[45]
Vascular endotherial growth factor	1.04 × 10^−9^–1.04 × 10^−10^	Si-ISFET	[46]
IgE	K_diss_ = 4.7 × 10^−8^	Graphene-ISFET	[47]
IgE	2.5 × 10^−10^	Carbon-FET	[48]
Interferon gamma	8.3 × 10^−11^	Graphene-ISFET	[49]
Platelet-derived growth factor	5 × 10^−12^	Carbon-FET	[50]
Platelet-derived growth factor	-	Diamond-FET	[51]

## Supplementary Materials

The following are available online at http://www.mdpi.com/1424-8220/18/1/49/s1, Table S1: Oligonucleotide sequences used in the work, Figure S1: Stem-loop structure for the FP predicted by Mfold web, Figure S2: Nondenaturating PAGE (photo without any modification) of PCR amplified (14 cycles) washout probes from magnetic beads modified with B1/Van_74, Figure S3: Nondenaturating PAGE (photo without any modification) of PCR amplified (14 cycles) washout probes from magnetic beads modified wirh DP/Van_74, Figure S4: Real time signal of the ISFET (in ∆ϕ) during Bsm DNA polymerase reaction in homogenous solution in Bsm buffer initiated (30–40 s) by addition of DP at different concentration (final concentration are marked on the picture), Figure S5: Real time signal of the ISFET (in ∆ϕ), Figure S6: Real time signal of the modified with Van_74 ISFET (in ∆ϕ, with background subtraction).
