# Supplementary material for: Amplified Detection of the Aptamer–Vanillin Complex with the Use of Bsm DNA Polymerase
Source: Sensors (Basel). 2017 Dec 26;18(1):49. doi: 10.3390/s18010049 (PMC5795474; doi:10.3390/s18010049)
Supplement: Supplementary file 1 [file sensors-18-00049-s001.pdf]

Supplementary Material

Amplified Detection of the Aptamer–Vanillin Complex with the Use of Bsm DNA Polymerase

Mariia Andrianova *, Natalia Komarova, Vitaliy Grudtsov, Evgeniy Kuznetsov and Alexander Kuznetsov

Scientific-Manufacturing Complex Technological Centre, 1–7 Shokin Square, Zelenograd, 124498 Moscow, Russia; nat.v.kom@gmail.com (N.K.); vitaliycatgrudcov@gmail.com (V.G.); kev@tcen.ru (E.K.); kae@tcen.ru (A.K.)

* Correspondence: smariika1987@gmail.com; Tel.: +7-(499)-734-45-21

Table S1. Oligonucleotide sequences used in the work. The underlined sequence is a capture sequence.

Sequence name	Sequence, 5′-3′ direction	Comment
B1_bank	CGACCAGCTCATTCCTCA-N10-GGAGTCTCGATG-N40-GGATCCGAGCTCACCAGTC	Library for SELEX
B1_biotin	Bio-GTC-Spacer18-**CATCGAGACTCC**	Capture probe with biotin
B1	**CATCGAGACTCC**	Capture probe without biotin
DP_biotin	biotin-GTC-spacer18-ACCA**CATCGAGACTCC**TGTGTCCTTT	Bsm dehybridization probe with biotin
DP	ACCA**CATCGAGACTCC**TGTGTCCTTT	Bsm dehybridization probe without biotin
Van_74	CGACCAGCTCATTCCTCAGGAGAAACATGGAGTCTCGATGATAGTAGGAGCGGCGGAACGTAGGAAGAGAGGATGACGGAGGATCCGAGCTCACCAGTC	aptamer
FP	FAM-TCTTGGFCFCAGGAGTCTCGATGTGGTATTGTGTCCAAGA-BHQ1	Fluorescence probe for Bsm DNA polymerase
PR	TCTTGGAC	Primer for Bsm DNA polymerase

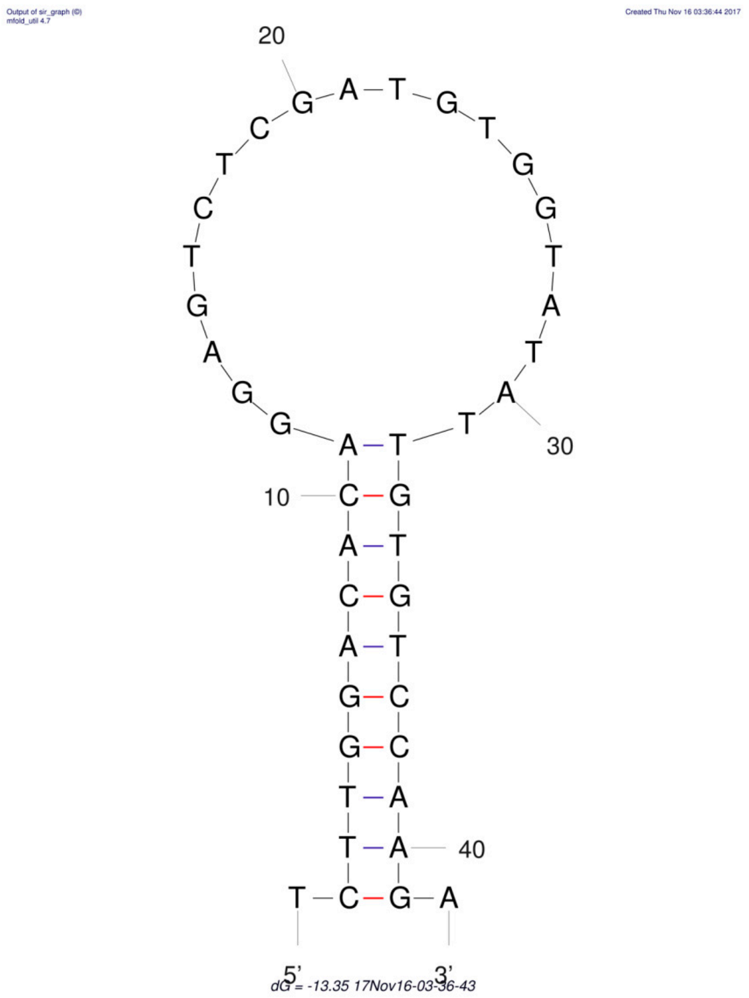

Figure S1. Stem-loop structure for the FP predicted by Mfold web (http://unafold.rna.albany.edu/?q=mfold).

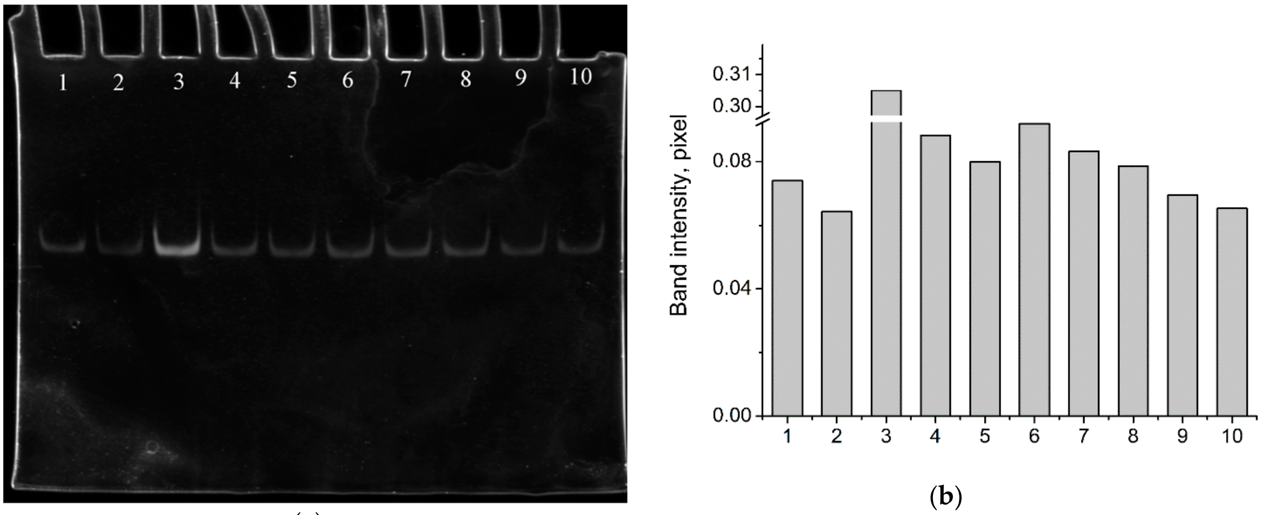

(**a**) (**b**)

Figure S2. Nondenaturating PAGE (photo without any modification) of PCR amplified (14 cycles) washout probes from magnetic beads modified with B1/Van_74 (**a**) and image analysis of phoresis with GelQuant.NET (**b**) for different incubation buffers: 1—Selection buffer with vanillin (1.5 mM), 2—Selection buffer, 3—Bsm buffer with vanillin (1.5 mM), this point doesn't correlate with other measurments, possible pipetting error, 4—Bsm buffer, 5—Bsm buffer with vanillin (1.5 mM), repeat, 6—Bsm buffer, repeat, 7—Bsm buffer with vanillin (1.5 mM), repeat, 8—Bsm buffer, repeat, 9—Selection buffer with vanillin (1.5 mM), repeat, 10—Selection buffer, repeat.

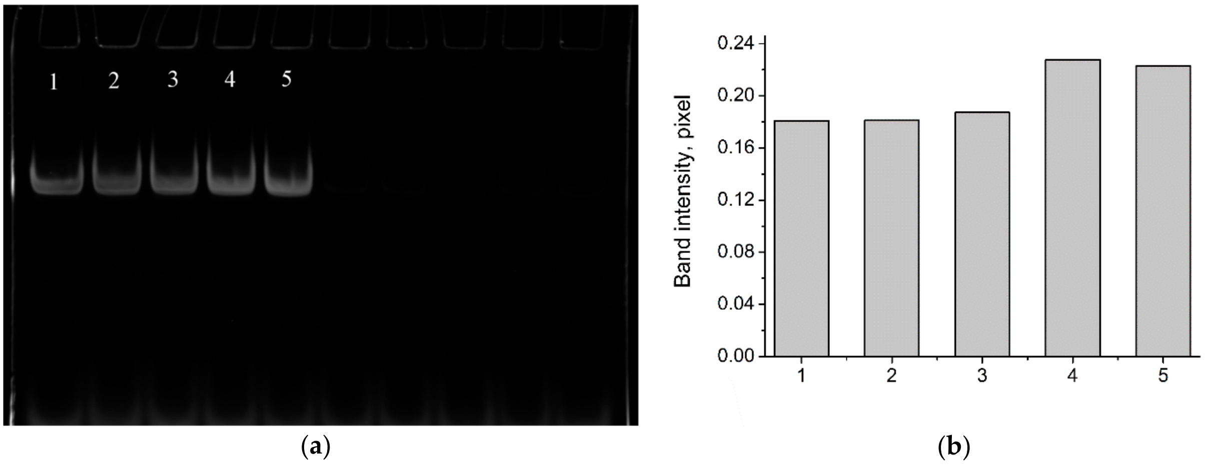

Figure S3. Nondenaturating PAGE (photo without any modification) of PCR amplified (14 cycles) washout probes from magnetic beads modified wirh DP/Van_74 (**a**) and image analysis of phoresis with GelQuant.NET (**b**) for different compositions: 1—Selection buffer, 2—Selection buffer, repeat, 3—Selection buffer, repeat, 4—Selection buffer with 1.5 mM of vanillin, 5—Selection buffer with 1.5 mM of vanillin, repeat.

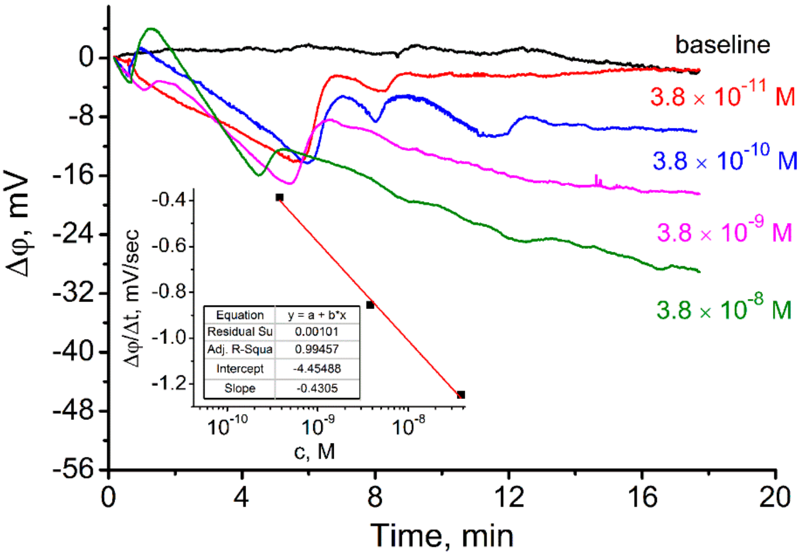

Figure S4. Real time signal of the ISFET (in Δϕ) during Bsm DNA polymerase reaction in homogenous solution in Bsm buffer initiated (30–40 s) by addition of DP at different concentration (final concentration are marked on the picture); Slope (Δϕ/Δt) dependence on DP concentration calculated from real time signal curves is presented as insert. Reaction conditions: 0.2 mM dNTP, 0.05 pmol/μl FP, 1.6 pmol/μl PR, 0.1 U/μl BSM DNA polymerase.

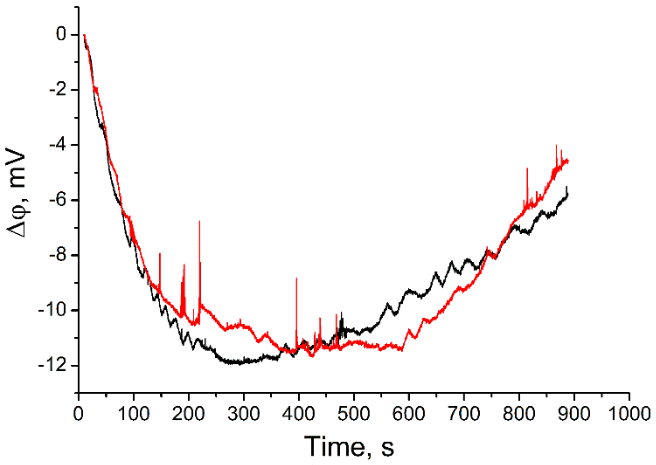

Figure S5. Real time signal of the ISFET (in Δϕ): black line—Selection buffer with 0.2 mM dNTP, 0.05 pmol/μl FP, 1.6 pmol/μl PR, 0.1 U/μl BSM DNA polymerase, red line—Selection buffer with 0.2 mM dNTP, 0.05 pmol/μl FP, 1.6 pmol/μl PR, 0.1 U/μl BSM DNA polymerase during polymerase reaction initiated by addition of DP (35 s) at concentration of 3×10^{-8} M.

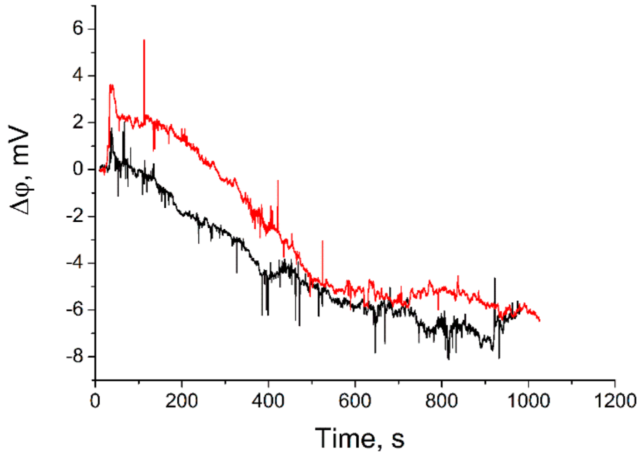

Figure S6. Real time signal of the modified with Van_74 ISFET (in Δφ, with background subtraction): black line—Selection buffer with 0.2 mM dNTP, 0.05 pmol/μl FP, 1.6 pmol/μl PR, 0.1 U/μl BSM DNA polymerase during polymerase reaction initiated by addition of DP (32 s) to final concentration 3×10^{-8} M, red line—Selection buffer with 0.2 mM dNTP, 0.32 pmol/μl DP, 1.6 pmol/μl PR, 0.1 U/μl BSM DNA polymerase during polymerase reaction initiated by addition of FP (35 s) to final concentration of 4.8×10^{-8} M.
